# Virtual Reality Support for Joint Attention Using the Floreo Joint Attention Module: Usability and Feasibility Pilot Study

**DOI:** 10.2196/14429

**Published:** 2019-09-30

**Authors:** Vijay Ravindran, Monica Osgood, Vibha Sazawal, Rita Solorzano, Sinan Turnacioglu

**Affiliations:** 1 Floreo Inc Washington, DC United States; 2 Celebrate the Children Denville, NJ United States

**Keywords:** autism spectrum disorder, interpersonal skills, virtual reality, instructional

## Abstract

**Background:**

Advances in virtual reality (VR) technology offer new opportunities to design supports for the core behaviors associated with autism spectrum disorder (ASD) that promote progress toward optimal outcomes. Floreo has developed a novel mobile VR platform that pairs a user receiving instruction on target skills with an adult monitor.

**Objective:**

The primary objective of this pilot study was to explore the feasibility of using Floreo’s Joint Attention Module in school-aged children with autism in a special education setting. A secondary objective was to explore a novel joint attention measure designed for use with school-aged children and to observe whether there was a suggestion of change in joint attention skills from preintervention to postintervention.

**Methods:**

A total of 12 participants (age range: 9 to 16 years) received training with the Joint Attention Module for 14 sessions over 5 weeks.

**Results:**

No serious side effects were reported, and no participants dropped out of the study because of undesirable side effects. On the basis of monitor data, 95.4% (126/132) of the time participants tolerated the headset, 95.4% (126/132) of the time participants seemed to enjoy using Floreo’s platform, and 95.5% (128/134) of the time the VR experience was reported as valuable. In addition, scoring of the joint attention measure suggested a positive change in participant skills related to the total number of interactions, use of eye contact, and initiation of interactions.

**Conclusions:**

The study results suggest that Floreo’s Joint Attention Module is safe and well tolerated by students with ASD, and preliminary data also suggest that its use is related to improvements in fundamental joint attention skills.

## Introduction

### Background

Autism spectrum disorder (ASD) is a heterogeneous neurodevelopmental condition characterized by variable degrees of impairment in social communication and restricted and repetitive patterns of behavior [1]. Prevalence rates of ASD have increased over time but show significant variability worldwide. In the United States, 1 in 40 children carries a diagnosis of ASD according to a 2016 survey of parents [2].

Although much attention has been paid to the pathogenesis and diagnosis of ASD, there remains a clear need for effective support for the core symptoms of ASD. Ideally, implementation of such supports during childhood will lead to optimal outcomes in adult life.

The economic impact of supporting individuals with ASD can be substantial for children with ASD. In the United States, the national cost of supporting children with ASD is estimated at US $61 billion in total [3], and in the United Kingdom, services and support are estimated at over £25 billion each year [4]. Special education expenses account for a large percentage of this estimate. Per year, the economic impact of supporting the health care and education needs of children with ASD has averaged more than US $17,000 per child. Students with ASD incurred higher school costs than their peers without ASD [5].

In addition to educational needs, students with ASD can require significant therapeutic support during their years in school. A survey of special education data noted that services included speech language therapy for 66.8% to 85.2% of autistic students, whereas 34.6% to 44.6% of students had behavioral services in place. This study noted that the significant number of students receiving speech language and occupational therapy was “consistent with the severity of communication impairments and with the pervasive effects of ASDs on activities of daily living” [6].

Optimal outcomes at as young as 8 years of age have been described for children with ASD who had initially been diagnosed before 5 years of age, with a percentage no longer meeting diagnostic criteria for ASD and having no significant differences in functional skills from peers without a history of ASD [7]. Individuals with such optimal outcomes were noted to have milder social symptoms than others who maintained a high-functioning ASD profile into young adulthood.

Children with ASD present with a range of social communication symptoms, including deficits in receptive and expressive language development, response to name, eye contact, appropriate use of gestures, and imitation skills. Joint attention, in particular, is a foundational social communication behavior that is often impacted during early development in children with ASD. Joint attention is a skill that involves responding to bids for attention as well as being able to initiate bids for attention. Older children with ASD who exhibit more developed language skills were noted to have shown better joint attention in early childhood [8]. Conversely, impairment in joint attention in early childhood is related to limited language development by school-aged children with ASD [9]. Joint attention is seen as a pivotal skill for the development of more advanced communication and social skills [10-13]. The ability to initiate joint attention in children with ASD is related to enhanced social interaction competence [14]. As such, joint attention has been studied as a target for interventions in children with ASD [11,15,16]

Given the increasing prevalence of ASD in the population, the impact of persisting problems in social communication, daily living skills, and the high societal costs associated with supporting individuals with ASD, it is critical, and timely, to develop innovative means of delivering opportunities for learning to affected children. Among a range of approaches, technology has been suggested and long researched (since the 1970s) as a potentially good fit for ASD populations. As such, and along with advances in virtual reality (VR) technology, head-mounted displays (HMDs) and virtual environments now offer new opportunities to design opportunities to target some of the core needs associated with ASD and promote progress toward optimal functional outcomes.

VR has been defined as a form of technology that presents a user with the opportunity to interact with computer-generated content while simultaneous engagement with the real world is limited [17]. This means that when a user puts on a pair of goggles, she will have the opportunity to see, hear, and manipulate an environment that is completely different from her real environment. When a virtual environment is created to meet the needs of a particular user, the user’s attention can be directed to specific elements by highlighting important information and filtering out extraneous information. Strickland described several elements of VR that were particularly relevant to meeting the needs of individuals with ASD, including a primary visual and auditory world that fits the typical learning preferences of this population, safe learning situations to repetitively practice daily living skills, and modification of the virtual environment to support generalization [18]. Immersive mobile VR utilizes a smartphone that is placed in an inexpensive headset and can provide an accessible and affordable experience. Furthermore, when the image that the user is seeing can be made visible on a tablet, an adult can provide monitoring, supervision, and coaching to support skill development. Virtual environments can provide engaging activities that cannot be offered in typical real-world therapy contexts, such as interaction with exotic animals or lessons that place users in scenarios that are difficult to replicate over multiple therapy sessions. This can serve as a powerful way to support learning in individuals with ASD.

VR has been actively studied for use in various aspects of health care, including health care provider training [19], pediatric pain distraction (eg, Smileyscope [20]), and stroke rehabilitation [21]. Its application has also been investigated to support mental health conditions, laying the groundwork for research on the potential benefit of VR for individuals with ASD.

Existing research compares VR exposure for social anxiety disorder with traditional in vivo exposure [22]. Both in vivo and “in virtuo” treatments were effective, but VR was much more practical for therapists. In another study, cognitive behavioral therapy (CBT) treatment for panic disorder that included a VR component was as effective as traditional CBT treatment, but the therapy that included VR required fewer sessions [23].

VR treatment was as effective as traditional CBT for treatment of arachnophobia in children [24], although there was some concern that children were more afraid of virtual spiders than real spiders. One way to address this concern is to let children know ahead of time what they are going to see in the virtual world, such as only characters of normal size, with no supernatural abilities.

Participants in a randomized controlled trial who received VR-based exposure therapy for posttraumatic stress disorder (PTSD) were helped more than those who received traditional PTSD treatments [25]. PTSD symptoms were measured using the Clinician-Administered PTSD Scale.

One reason for considering VR as an approach is in part due to positive results from previous research—they have shown promising outcomes. Researchers at Politecnico di Milano have undertaken a pilot study using supervised low-cost VR via Google Cardboard on a small sample of 5 children with varied developmental disabilities including ASD [26,27]. The results have been promising. The children in the study accepted the Google Cardboard headset, and therapists found the therapy easy to use and beneficial to their clients. The therapeutic content is a storytelling app that requires the user to maintain eye contact with a virtual character for the story to continue, thus developing attention and engagement skills.

In another pilot study, 29 adults with ASD used an HMD (Oculus Rift), first for 10 min, and then possibly for a longer session at a later date [28]. Although the content was not therapeutic, it was entertaining and offered similar physical effects to the proposed theoretic content. A recent systematic review by Bradley and Newbutt noted the limited scope of existing research and the need for more robust ongoing investigation in the potential of VR HMDs for learning in individuals with ASD [29].

Another recent study asked 3 children (aged 10-13 years) with ASD using an HMD to improve social understanding and social skills [30]. These participants used the VR-based therapy once per week for 6 weeks. All subjects completed all sessions, and therapists report that the treatment modality was motivating. All subjects showed improvement with regard to targeted behaviors at the end of the study.

In all VR-based therapies, there is the potential for unwanted physical side effects from being in a virtual environment. These side effects are similar to motion sickness or simulator sickness and are often called cybersickness. Symptoms include dizziness, nausea, eye strain, and fatigue. Best practices for clinical trials involving VR-based therapy include informing users about potential risks, monitoring users as they use VR, informing users how to minimize side effects, and designing VR environments to prevent as many causes of sickness as possible.

In practice, VR therapy for anxiety disorders involves limited amounts of VR exposure spread out over a suitable length of time, and side effects are not a problem for most patients. However, it should be noted that individuals with autism often have comorbid sensory processing disorders, which can increase or decrease the likelihood of unwanted side effects. Newbutt et al found that only 4 of their 29 participants with ASD dropped out due to cybersickness [28].

To better understand the ability of children with ASD to tolerate VR using modern HMDs and to assess for the occurrence of adverse effects related to VR use, the authors developed an exploratory study evaluating the safety and feasibility of the Floreo VR platform.

### Objectives

The primary objective of this pilot study was to determine feasibility for using the *Floreo Joint Attention Module* to support joint attention skills in a VR setting in students with ASD attending a special education school. Safe use, potential adverse effects, and tolerability of Floreo’s VR software by school-aged participants were of particular interest in conducting this pilot study. A secondary objective was to explore the changes in participant joint attention skills over time by using a novel joint attention assessment for school-aged children. This was an open-label pilot study with no control group.

## Methods

### Participants

Eligible participants were recruited from the student population at Celebrate the Children school. Celebrate the Children is a private special education school. The school’s mission highlights the use of state-of-the-art interventions to support the learning needs of children with social and communication challenges such as seen in ASD. Because this pilot study focused on the feasibility of using the Floreo Joint Attention Module as an educational support and as an element of the normal summer camp curriculum at a special education school, it was deemed to be exempt from review by an Institutional Review Board. Instead, the initial pilot proposal was reviewed by an outside consultant with direct feedback incorporated in an edited and updated version of the protocol that was used for the pilot study.

Potential participants were identified by Celebrate the Children staff. Families were sent a SurveyMonkey questionnaire to collect demographic and health information to determine eligibility based on inclusion and exclusion criteria. SurveyMonkey, which was also used to collect pre- and postsession information throughout the pilot, is Health Insurance Portability and Accountability Act (HIPAA) compliant for confidential management of protected health information.

Inclusion criteria consisted of an age between 7 and 18 years and diagnosis on the autism spectrum (or any diagnosis of ASD, autistic disorder, Asperger syndrome, pervasive developmental disorder, or pervasive developmental disorder, not otherwise specified). Exclusion criteria were history of seizures or known photosensitive response on electroencephalogram, migraines, vertigo or other serious balance disorder, or psychosis or other disorder disrupting the ability to distinguish reality from nonreality. In addition, families were also asked about their child’s expressive communication level, augmentative and alternative communication techniques, prescribed and over-the-counter medications used, vision screening, problems with vision, use of corrective lenses, and history of problems with VR and 3D entertainment (Table 1).

Participants ranged from 9 to 16 years of age. Consistent with broader ASD demographics, 10 of the 12 participants were male. Nearly half of the participants were described by their caregivers as pre- or nonverbal, and another third were described as minimally verbal. Three-quarters of participants made use of some form of alternative augmentative communication (Table 2). All subjects were able to follow simple verbal directions.

**Table 1:** Characteristics of pilot study participants (N=12).

Characteristics		Value
Age (years), mean (range)		13.5 (9-16)
Overall participation, n (%)		12 (100)
**Gender, n (%)**		
	Male	10 (83)
	Female	2 (17)
**Caregiver-rated verbal skills, n (%)**		
	Verbal	3 (25)
	Minimally verbal	4 (33)
	Pre- or nonverbal	5 (42)
**Alternative augmentative communication used, n (%)**		
	Yes	9 (75)
	No	3 (25)
**Medication use, n (%)**		
	Yes	6 (50)
	No	6 (50)
**Corrective lenses, n (%)**		
	Yes	5 (42)
	No	7 (58)
**EEG^a^** **performed in the past, n (%)**		
	Yes	7 (58)
	No	5 (42)

^a^EEG: electroencephalogram.

**Table 2:** Forms of augmentative and alternative communication used (N=12).

Communication approach	Value, n (%)
Pictures or symbols	3 (25)
Communication book or board	2 (17)
Electronic communication	7 (58)
AAC^a^ app	2 (17)
Keyboard or letterboard	3 (25)
American sign language	1 (8)
Picture exchange communication system	1 (8)
Facilitated communication	4 (33)
Rapid prompting	3 (25)

^a^AAC: augmentative and alternative communication.

Of note, for 2 of the participants, baseline responses indicated that the participant regularly took prescribed or over-the-counter medication and that the participant would be on the same medication schedule over the summer, but details were not provided on the specific medications taken.

For informed consent procedures, parents of eligible participants were sent consent and assent forms via Docusign. A phone call was arranged to discuss the study with a parent and the eligible participant, to answer any questions and to obtain the participant’s assent. Parents and eligible participants sent back signed consent forms through the confidential Docusign process. Assent was obtained in person by study staff during the initial study visit. In addition to the consent and assent forms, families were also provided a child-oriented brochure, in comic book form, about VR, how to experience it, and possible side effects.

To explore this pilot study’s primary objective of the feasibility of using Floreo’s Joint Attention Module with school-aged children with ASD, data collection focused on reports from school staff serving as monitors for the study participants during the VR sessions. In addition, evidence of physical side effects, discomfort, and distress was reported as well by monitors to explore the safe use of Floreo’s module. The secondary objective of the potential impact of the Floreo VR experience on participants’ joint attention skills was assessed through a novel play-based behavioral assessment, as described in further detail below.

### Teaching Approach

**Figure 1:**
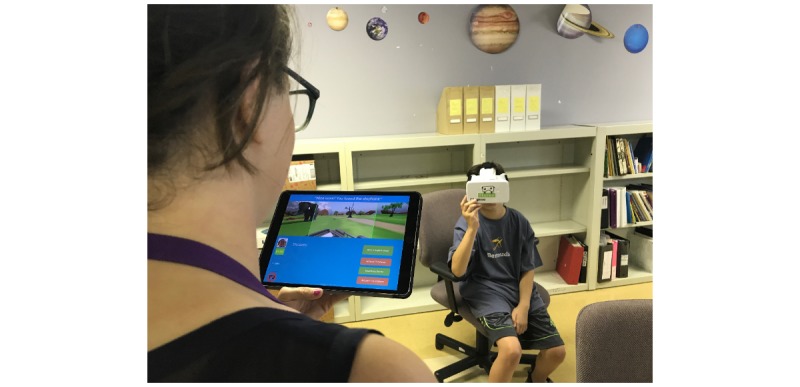
The monitor uses an iPad to supervise the Floreo session with a learner.

The Floreo Joint Attention Module is software that offers a supervised VR experience for individuals with ASD. Floreo’s module provides a 3D immersive scene for Google Cardboard–compatible smartphones and a supervisory overview that can run on smartphones or tablets. To use the software, a monitor, who can be a therapist, teacher, or parent, places the smartphone into a Google Cardboard–compatible VR HMD and then assists the individual with ASD in putting on the HMD. The monitor guides and supervises the user on a device (tablet or smartphone) paired with the user’s device over a network connection (Figure 1).

**Figure 2:**
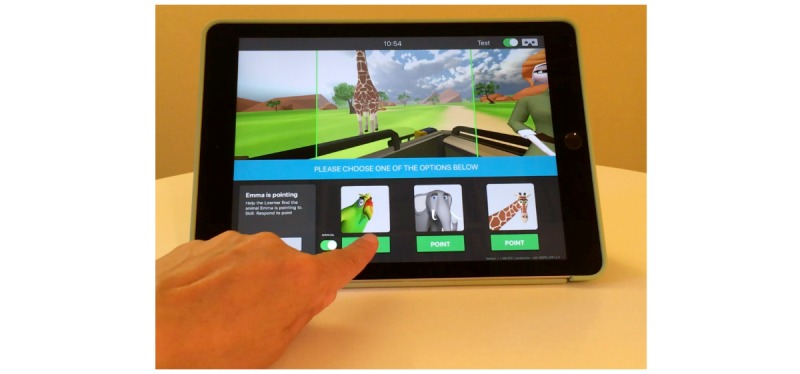
An example of the monitor’s view of a Joint Attention Module learning card.

The instructional content consists of a Joint Attention Module including separate learning cards addressing specific subskills necessary to develop appropriate joint attention behaviors. Each learning card consists of a VR environment in a safari-themed setting, complete with animals designed to draw the student’s attention when necessary. Users proceed through learning cards sequentially to achieve the set goals related to the demonstration of target joint attention subskills. An avatar in the virtual environment initiates and responds to joint attention bids and can also verbally prompt the user when needed to make further progress through a learning card to achieve goals. The monitor tracks the student’s engagement and progress through each learning card and provides redirection and feedback as necessary. The monitor’s display provides a broader view of the learner’s display along with a control panel that allows the monitor to select options and guide the learner through a lesson (Figure 2; a video of the monitor view of the Joint Attention Module is presented as Multimedia Appendix 1).

In this pilot study, the Floreo Joint Attention Module was implemented in a special education setting by a school staff member working with a student during summer camp.

Participants were engaged in 1 to 2 VR episodes per session. Each VR episode lasted no more than 5 min, with a break in between the episodes for at least 3 min. Sessions occurred 3 days per week, with at least 48 h between sessions. A total of 14 VR sessions were completed over a 5-week period.

Floreo’s team members conducted training with designated monitors in preparation for the pilot study. This was done on-site at Celebrate the Children school and included a presentation by Floreo’s team members as well as opportunities to practice using Floreo’s platform with feedback from Floreo’s staff. Objectives of the pilot study, benefits of targeting and training joint attention skills, content of the Joint Attention Module Learning Cards, detailed instructions on the use of the Floreo platform, and the overall structure of the curriculum including pre- and postsession questionnaires were covered in the on-site presentation.

Prior to the first VR session, all participants underwent a joint attention assessment to capture their existing status of joint attention behavior. This assessment was a novel measure developed by the study team to directly assess joint attention skills in school-aged children (details of the assessment are available from the authors upon request).

A review of the joint attention and social communication measure literature revealed several challenges with the incorporation of existing measures in intervention research. At a high level, a recent review of social communication behavioral measures that might be used for treatment endpoints in ASD found that there were no measures appropriate to use without conditions [31]. Some of the measures reviewed were initially developed as screens for ASD-associated behaviors, such as the Social Responsiveness Scale) and the Autism Spectrum Rating Scales. Other measures included in the review are broad in scope, assessing either adaptive behaviors as a whole or a range of ASD-related behaviors, such as the Vineland Adaptive Behavior Scales, the Adaptive Behavior Assessment System, the Pervasive Developmental Disorder Behavior Inventory, and the Autism Diagnostic Observation Schedule (ADOS). The Communication and Symbolic Behaviors Scale (CSBS) and the Early Social Communication Scales are only appropriate for use in infants and toddlers, or young children with delayed communication skills. The CSBS in particular is only normed up to 2 years of age.

Another recent review of approaches to assessment of minimally verbal school-aged children with ASD found that measures addressing intentional communication “required high levels of expertise to code and interpret” or were “informal and nonstandardized” [32].

Of note, the Brief Observation of Social Communication Change has been developed based on social communication behaviors rated in the ADOS, and research is being conducted on its utility in clinical trials [33].

Bean and Eigsti published a joint attention measure for school-aged children and adolescents [34], but the elements of this measure did not map well with the objectives of our Joint Attention Module. In addition, there had been no further research on this scale at the time, and so the team made the decision not to use this particular measure.

For purposes of this initial pilot study, the team instead decided to develop a novel joint attention assessment that could be administered quickly, included play-based activities appropriate for school-aged children, and focused on the key joint attention behaviors targeted in Floreo’s VR Joint Attention Module. This measure was modeled after the joint attention measure found in the CSBS [35]. It was geared toward teenage students with limited verbal skills and needs in social reciprocity. The measure was designed to measure the instances of joint attention (specifically shifting eye gaze between a toy and a play partner) and used age-appropriate appealing toys (cause and effect and sensory toys as well as one that allows for turn taking games). It should be noted that while this measure assessed joint attention, this skill does not exist in a vacuum, but as a component of a socially reciprocal interaction, and so the assessment included other features of social reciprocity. In scoring the measure, the team looked for instances of social reciprocity (initiating, responding, continuing conversation beyond 2 turns, commenting, questioning, requesting, protesting, and refusal), response to greeting, shifting eye gaze in response to a point, and instances of prolonged eye contact toward a person or an object. Notes were taken on affect and mood during video review.

This joint attention assessment was then repeated by the same examiner, a speech language therapist, 4 weeks after VR sessions had concluded.

**Figure 3:**
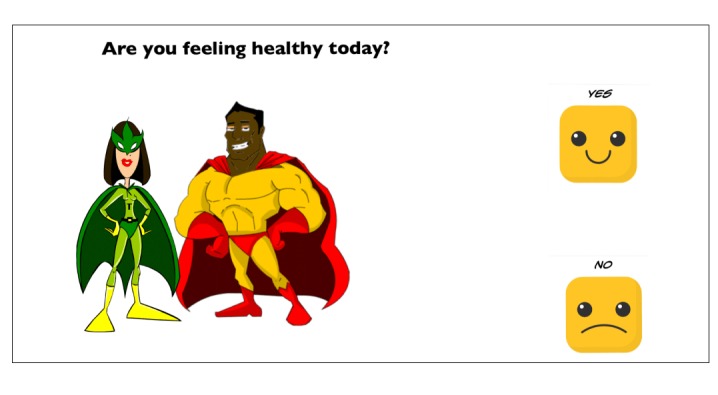
A question from the Pre-Session Check with written and visual components.

All VR sessions began with a greeting by the monitor, an employee of Celebrate the Children who was trained by the Floreo staff to use the Joint Attention Module. Participants then completed a SurveyMonkey questionnaire, the “Presession Check,” with written and visual components that inquired about general health status, balance, sleep, and interest in continuing with Floreo (as a means of verifying consent to participate in the Floreo app experience; sample image shown in Figure 3). The monitor then set up the Floreo system on an iPad used by the monitor and a phone used by the participant inside the HMD. The HMD was cleaned if necessary. The participant then put the HMD on and the monitor checked that the HMD had been put on correctly.

The first session consisted of 1 learning card episode to help participants orient themselves to the VR environment. At subsequent sessions, participants were given the opportunity to engage in 1 or 2 VR episodes per session. Investigators were told to stop therapy if the participant appeared to be experiencing or reporting any side effects. Prompts for side effects included preference for looking at the corners of the screen, change from standing to sitting or vice versa, fidgeting, changes in breathing pattern, sweating, verbalizing their distress (if possible), or holding hands to the head. Participants proceeded through a consistent schedule of Joint Attention Module sessions (Table 3).

In general, monitors were encouraged to have participants progress through learning cards in a sequential manner, from Learning Card 1 to Learning Card 6, but they were given the flexibility to adapt the learning card sequence as deemed appropriate for individual participants’ needs. For example, if a participant seemed to get more frustrated with the demands of a given learning card, the monitor could return to an earlier learning card for the next episode. In this particular study, the monitors maintained the recommended schedule of sessions to support participant progress through learning cards.

**Table 3:** Joint Attention Module experience schedule.

Session number	Learning card number	Learning card name
Session 1	Learning Card 1	Explore
Session 2	Learning Card 2	Who made that sound?
Session 3	Learning Card 3	Watch me go
Session 4	Learning Card 3	Watch me go
Session 5	Learning Card 4	Emma is pointing
Session 6	Learning Card 4	Emma is pointing
Session 7	Learning Card 4	Emma is pointing
Session 8	Learning Card 5	Emma wants to look too
Session 9	Learning Card 5	Emma wants to look too
Session 10	Learning Card 5	Emma wants to look too
Session 11	Learning Card 6	Get Emma’s attention
Session 12	Learning Card 6	Get Emma’s attention
Session 13	Learning Card 6	Get Emma’s attention
Session 14	Learning Card 1 plus 1 Learning Card of the participant’s choice	Explore plus any of the above

**Figure 4:**
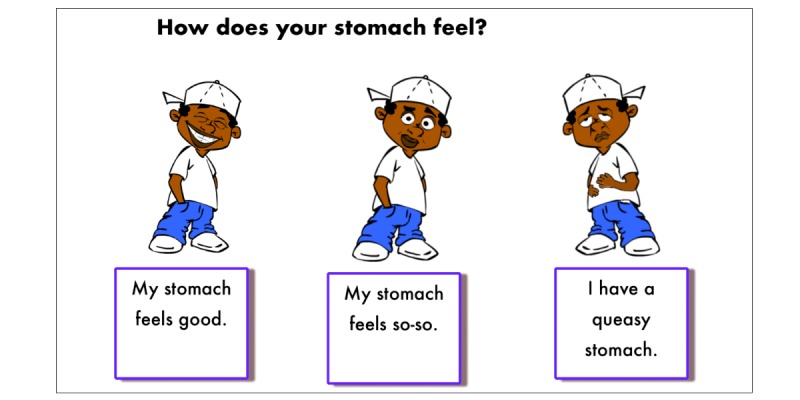
A question from the Post-Session check with written and visual components.

After each session, participants completed a SurveyMonkey questionnaire, the “Postsession Check,” with written and visual components that inquired about the level of alertness, eye discomfort, clarity of vision, headache, stomach ache, balance, and enjoyment of having used Floreo platform (sample image shown in Figure 4). The monitor also completed a SurveyMonkey questionnaire asking about participant’s tolerance of the HMD, perceived enjoyment of the VR session, any indication of negative side effects, and perceived value of Floreo VR sessions for the participant. (Survey questions are available from the authors upon request.) The questions addressing tolerance, enjoyment, negative side effects, and value of the Floreo experience offered “Yes” and “No” answer choices. The final question on the monitor survey was a request for qualitative feedback on the participant’s experience and any additional information related to the VR session.

In addition, a short, simple hand-eye coordination activity was offered to participants after the VR session to support reorientation of their eyes to the real world. Participants then returned to their regularly scheduled day camp activities.

Data collected throughout the study was reviewed daily by study staff to explore the safe use and acceptance of the headset and Floreo VR experience by study participants.

## Results

### Virtual Reality Session Feedback

Overall, 14 Floreo VR Joint Attention Module training sessions were conducted over a 5-week period. Participants attended 80.3% (135/168) of sessions. Participants were able to complete 97.6% (164/168) of VR sessions attended. Incomplete sessions only occurred on the first day of the pilot, and afterwards school staff introduced acclimating strategies that helped ease the participants into the VR sessions, so all participants were able to complete sessions for the remainder of the study.

With regard to monitor survey data, a number of surveys were not completed because of participants’ absence during the pilot study period. One participant lost his glasses after the first 5 sessions, and, after a discussion between Celebrate the Children personnel and the Floreo team after 2 sessions of variable participation, he was not permitted to continue using Floreo VR for the final 7 sessions. In total, between participants’ absence and missing monitor surveys, the Floreo team received 79.1% of the total possible number of surveys (133 sessions with completed monitor surveys compared with 168 total sessions conducted).

In summarizing monitor survey responses, several questions were left incomplete on postsession monitor surveys, affecting totals for the 4 key safety and usability questions.

Monitor surveys were analyzed to determine the percentage of “Yes” responses out of the total number of surveys received across all sessions (Table 4 **)**. Results indicated that 95% (126/132) of the time participants tolerated HMD use. Participants were rated by monitors as seeming to enjoy Floreo VR 95% (126/132) of the time. Negative side effects were described 9% (12/129) of the time. Ill effects that were described in open comments included participants bothered by the headset on 8 occasions, restlessness on 7 occasions, eye rubbing on 2 occasions, and fatigue on 1 occasion. Of note, 1 participant experienced 4 of the episodes of restlessness and another participant experienced 2 of the other restlessness episodes. In addition, 1 participant experienced 4 of the “bothered by headset” episodes, and another participant experienced 2 of the other “bothered by headset” episodes. Monitors rated Floreo VR sessions as valuable for participants 96% (128/134) of the time.

**Table 4:** Monitor survey responses.

Postsession monitor survey question	Surveys received (N)	Response (yes), n (%)
Did the participant tolerate wearing the headset today?	132	126 (95)
In your opinion, did the participant enjoy their VR^a^ session?	132	126 (95)
Did you notice any signs of negative side effects?	129	12 (9)
So far, do you think the Floreo sessions have been valuable to the participant?	134	128 (96)

^a^VR: virtual reality.

Despite the challenges some participants faced tolerating the HMD and VR experience on the first day of the study, following the acclimatization procedures implemented by Celebrate the Children personnel no participants dropped out of the study because of either intolerance of the HMD or VR environment, or secondary to side effects.

Participants completed the presession surveys at a 100% rate, and the postsession surveys showed a 98% completion rate. However, upon review and comparison with monitor survey responses, participants’ responses were determined to be inconsistent that led the team to question the reliability of the responses.

Qualitative monitor feedback was positive based on survey results during the study and also in a poststudy debriefing. Monitors noted progress in participants’ ability to utilize the app and increased comfort with the equipment. Monitors also saw the Floreo platform as having “a real potential to help our kids on the spectrum...definitely something that kids need...”

### Joint Attention Assessment

Each participant’s pre- and post-Floreo joint attention assessments were recorded. Video recordings for individual participants were later reviewed and coded by the Floreo team’s speech language therapist to document the number of occurrences of specific joint attention behaviors. These behaviors included the following: looks at activity/object; shift eye gaze; initiate requests; respond to requests; participant initiates interaction; participant responds to interaction; direct eye contact (participant initiated); follows a point; and points.

Lack of eye contact could not be attributed to lack of engagement with the activities in most cases. Overall, all the participants were either highly engaged or intermittently engaged with one or more of the activities. If a participant showed a lack of interest in one of the activities, it was abandoned for another activity. The level of involvement of 2 of the participants decreased somewhat during the posttest. In both of these cases, the participants were reported to have had some difficulty readjusting to the school setting. Additionally, the posttest for 1 participant was shortened to about 6.5 min as the individual appeared to be experiencing some anxiety during the assessment. In one instance, the participant seemed to become somewhat more engaged in the activities during the posttest.

In evaluating the results of the joint attention assessment, a meaningful difference was determined to be a change of more than 2 instances of a behavior between the pretest and the posttest.

Analysis of the pre- and post-Floreo joint attention assessment results showed that 10 out of the 12 participants demonstrated improvement in 1 or more key areas (total number of interactions, initiating interactions, and eye contact; Table 5).

**Table 5:** Change in scores of key behaviors on the joint attention assessment of pre- and post-Floreo Joint Attention Module experience.

Participant	Total number of interactions			Use of eye contact			Initiation of interactions		
	Prestudy (n)	Poststudy (n)	Change	Prestudy (n)	Poststudy (n)	Change	Prestudy (n)	Poststudy (n)	Change
1	9	13	4	1	0	−1	1	0	−1
2	10	14	4	1	2	1	2	1	−1
3	17	14	−3	8	1	−7	1	3	2
4	47	54	7	21	37	16	9	10	1
5	18	24	6	3	0	−3	4	6	2
6	43	76	33	22	57	35	9	12	3
7	14	14	0	0	2	2	5	4	−1
8	10	30	20	2	20	18	0	8	8
9	29	47	18	14	32	18	11	35	24
10	25	20	−5	4	10	6	6	11	5
11	24	32	8	20	27	7	0	6	6
12	23	29	6	10	16	6	13	12	−1

All interactions that were associated with specific types of communicative intents were counted and divided into 4 groups depending on whether the interaction included associated eye contact and/or intentional verbal/vocal components. The 4 groups included the following: interaction with eye contact and a verbal/vocal message, interaction with eye contact but without accompanying verbal message, interaction without eye contact but with verbal/vocal message, and interaction without eye contact and without verbal vocal message. Analysis indicated that 9 of the 12 participants (75%) showed an increase in the number of total interactions from pretest to posttest.

Another key behavior assessed by the team was eye gaze shift between an item or event of interest and a communication partner, outside of any other communicative act (such as requesting, gaining attention, responding). Of the 12 participants (58%), 7 demonstrated an increase in eye contact during interactions from pretest to posttest, and 4 of these individuals (33%) showed a pronounced increase in eye contact.

In addition, 5 of the 12 participants (42%) demonstrated an increase in instances of initiation of interactions from pretest to posttest.

In general, no meaningful increase was observed in requesting or in responding to requests between pre- and posttest.

## Discussion

### Principal Findings

In this study, Floreo collaborated with the special education school Celebrate the Children to collect pilot data on the feasibility and safety of using Floreo’s mobile VR platform for training joint attention skills in children with ASD. In addition, the pilot data obtained on a novel joint attention measure designed for use in school-aged children with ASD suggests that training with Floreo’s Joint Attention Module was related to improvements in social reciprocity skills in these children. Findings from this pilot study support ongoing research on the practical use of this platform and on the effectiveness of the joint attention training content on social communication skills in ASD.

Floreo’s intervention is an immersive mobile VR system designed to support the development of fundamental social communication skills in individuals with ASD. Other research teams have studied the use of nonimmersive VR systems on social skills in individuals with ASD [36] or have used immersive VR to target daily living skills through the use of social story–inspired systems [26].

The research team also identified several key issues related to using VR to support the developmental needs of children with ASD. One concern raised frequently by professionals and researchers was the ability of individuals with ASD and sensory sensitivities to tolerate wearing the HMD and remaining engaged in a VR experience. On the first day of the pilot study, several participants had difficulty completing the session. As a result, Celebrate the Children personnel implemented acclimating strategies to help ease participants into the VR sessions, and all participants were subsequently able to complete the remainder of attended sessions. No participants dropped out of the study because of inability to tolerate the use of the VR headset or participation in VR training sessions. One participant lost his glasses, and study staff determined that he should not continue in further sessions because of the fact that the participant might be at a greater risk for eye discomfort and headache without his corrective lenses. Owing to both the hypersensitivities experienced by some individuals with ASD and medical comorbidities such as gastrointestinal symptoms and seizures, there were additional concerns about the health and safety issues associated with the VR experience. Side effects were noted in less than 10% of sessions. Two participants in particular had a higher incidence of side effects that included restlessness and appearing to be bothered by the headset.

Another important element of this study was the successful implementation of pre- and post-VR session checks associated with the app itself. The presession check-in questionnaire can be used to gauge existing medical symptoms that might have a negative impact on the user’s VR experience, as well as to confirm readiness to engage in the VR experience. The postsession questionnaire serves to capture symptoms that may have developed as a result of VR use, as well as to rate the user’s level of engagement and enjoyment of the VR session. In surveys completed after working with participants, monitors at Celebrate the Children provided positive feedback regarding the feasibility of using Floreo’s Joint Attention Module. In a high percentage of sessions, monitors reported that participants were able to tolerate the VR headset and seemed to enjoy using Floreo VR, and monitors also responded that the VR experience was valuable for participants.

A secondary objective of this study was to evaluate the feasibility of using a novel joint attention measure to rapidly assess the status of participants’ joint attention skills before and after the VR support program. As noted above, there are no widely used measures available for focused assessment of joint attention skills in school-aged children. As improvement in this particular set of skills is the ultimate goal of using the Joint Attention Module in children with ASD, the study team wanted to begin an exploration of the effectiveness of this support in the target population. A review of the video-recorded pre- and postprogram joint attention measures suggested that conducting 14 sessions of VR-based joint attention skill training over 5 weeks was related to a higher total number of social interactions, more eye contact during interactions, and more episodes of initiation of interactions on the part of participants.

### Limitations

Several limitations should be recognized in this pilot study. First, the study team did not compare the experience of using Floreo’s VR Joint Attention Module with a control group of same-age peers receiving typical types of support at the school’s summer camp or no specific social communication support. The primary objective of this pilot study was to explore the safety and feasibility of the use of Floreo’s module by the target learner population, and further research was planned to specifically evaluate the effectiveness of the Floreo platform on social communication skills that will incorporate a control condition. Second, although the study team attempted to elicit direct feedback from participants after each session, questionnaire responses were not consistent with what was noted by monitors or other observers. In addition, many participants’ limited communication skills impacted their ability to independently complete the surveys. Therefore, the team focused on monitor feedback to generate an impression of the participant experience of using Floreo’s module. On the basis of the paucity of published research on joint attention measures for school-aged children available at the time of initial study planning (as described in greater detail in the Methods section), the team developed a novel joint attention assessment for use in this study. Since the completion of this pilot study, new research has been published providing further support for the use of the Joint Attention Protocol in school-aged children [37], and the team plans to use this measure in future research studies. In addition, the study team will consider more optimal approaches to ensuring independence and validity of usability responses in participants with limited communication skills or mild-to-moderate intellectual disability in future research study design.

### Conclusions

Overall, the results from this pilot study are extremely promising for the potential of Floreo’s module to be well received and used by individuals with autism and the therapists, teachers, and parents working with them. This introduces a new and innovative mechanism for providing support for social communication skills in individuals with ASDs. The long-term vision is for the Floreo VR platform to be used to develop a diverse set of lessons designed to teach a variety of skills in individuals with autism and other developmental disabilities. The Floreo app, designed to be used with the smartphones and tablets that are already accessible in many homes, classrooms, and therapists’ offices, along with a low-cost HMD, can help reduce the costs and facilitate access to support for individuals with ASD and their caregivers.

## Appendix

Multimedia Appendix 1 Video of the monitor view of the Joint Attention Module.

## Abbreviations

ADOS: Autism Diagnostic Observation Schedule
ASD: autism spectrum disorder
CBT: cognitive behavioral therapy
CSBS: Communication and Symbolic Behaviors Scale
HMD: head-mounted display
PTSD: posttraumatic stress disorder
VR: virtual reality
